# Effectiveness of the Workshop “Adolescent Depression: What Can Schools Do?”

**DOI:** 10.3389/fpsyt.2015.00067

**Published:** 2015-05-08

**Authors:** Vania Martínez, Daniel Espinosa, Pedro Zitko, Rigoberto Marín, Sara Schilling, Camila Schwerter, Graciela Rojas

**Affiliations:** ^1^Centro de Medicina Reproductiva y Desarrollo Integral del Adolescente (CEMERA), Facultad de Medicina, Universidad de Chile, Santiago, Chile; ^2^Millennium Institute for Research in Depression and Personality, Santiago, Chile; ^3^Unidad de Estudios Asistenciales, Complejo Asistencial Barros Luco, Santiago, Chile; ^4^Facultad de Medicina, Universidad Diego Portales, Santiago, Chile; ^5^School of Medicine, Facultad de Medicina, Universidad de Chile, Santiago, Chile; ^6^School of Public Health, Facultad de Medicina, Universidad de Chile, Santiago, Chile; ^7^Department of Psychiatry and Mental Health, Clinical Hospital, Universidad de Chile, Santiago, Chile

**Keywords:** adolescent depression, depression education, educational program, schools, school staff

## Abstract

**Introduction:**

Adolescent depression is associated with serious consequences. School staff is in a unique position to screen and refer adolescents with depression in a timely manner, and can collaborate with healthcare teams to assist in the proper management of the disease. The objective of this paper is to describe the results of a workshop that aims to improve the knowledge of adolescent depression among school staff.

**Materials and methods:**

This was a single-arm trial with a pre-post design. Six workshops were conducted in four cities in Chile. Each workshop lasted 4 h. Participatory methodology was used. A 26-item knowledge questionnaire about adolescent depression, with the alternatives “I agree,” “I disagree,” and “I don’t know,” was administered to the participants, before and after the workshop.

**Results:**

A total of 152 people participated in the trial. Of these, 74.3% were female, and 44.7% were school psychologists, 25.0%, teachers, 17.8%, school counselors, and 5.3%, social workers. On average, there were 69.6% (SD 21.3) correct responses on the initial test, and 91.8% (SD 8.0) on the final test. All items had an increase of correct answers and a decrease of “don’t know” answers. There were notable increases of correct responses on statements dealing with myths: “Antidepressants for the treatment of depression in adolescents must be avoided because they produce dependence” (59–96%), and “Depression in adolescence is better defined as a weakness of character than as a disease” (75–95%). School psychologists scored higher than the other participants on the questionnaire both before and after the workshop.

**Conclusion:**

The workshop: “Adolescent depression: What can schools do?” can improve school staff knowledge of this topic, especially aiding to dispel myths regarding the disease and its treatment. This can help bring about timely case detection and improved collaboration with health team for proper handling of adolescent depression.

## Introduction

Depression is a highly prevalent and recurrent illness that affects people of all ages and carries high individual, family, and social costs (1). Depression in adolescents is of particular importance – prevalence begins to increase in this period (2) and is associated with persistent and considerable functional impairment and serious negative consequences, such as suicide, poor performance at school, difficulty with interpersonal relationships, risk-taking behavior, and physical health problems (2–4).

In developed countries, the point-prevalence of depression in adolescents is estimated to range between 4 and 8% (5). The lifetime prevalence of having had at least one depressive episode reaches 10% at 16 years of age (6), and more than 20% at 18 years of age (7). Even more adolescents have sub-threshold depressive symptoms. Those symptoms also have negative consequences and are a risk factor for later depression and suicidal behavior (7, 8). Furthermore, most adults with recurrent depression began having depressive episodes as adolescents (9).

In an epidemiological study carried out in Chile, 8.3% of adolescents between the ages of 12 and 18 were found to be suffering from major depression. The same study reported a huge gap between the adolescent population who manifest some form of mental health disorder and those who finally receive treatment (10).

Adolescents, in general, are less inclined to seek medical treatment for causes related to morbidity, and this tendency is even greater when the problems are related to mental health (11). School staff is in a unique position to screen, refer, and motivate their adolescent students in the early stages of depressive episodes. They also can collaborate with healthcare teams to properly manage adolescent depression.

Brief training offers a potentially cost-effective format for disseminating knowledge of adolescent mental health among school staff. Some studies have found that brief training can generate significant improvements in school staff knowledge. This has been investigated in suicide (12–14), ADHD (15–17), mental health first aid (18), and deliberate self-harm (19) with good results.

Training sessions with school staff could also improve their ability to identify and support students with depression, though little research has been conducted to evaluate this. A study from Scotland failed to show the effectiveness of a school-based psychoeducational intervention designed to help teachers recognize the symptoms of clinical depression in their adolescent students (20), despite promising results in the pilot study (21). The authors of that study suggested that one explanation of the negative results is that some of the teachers were reluctant to adapt a medical perspective of adolescent depression, as they viewed depression as having more of a social or “moral component” from their experiences in the classroom (20).

In light of this experience, we designed a workshop for a multi-disciplinary group of school staff (not only for teachers but also for psychologists, counselors, social workers, and administrators), so that the perspectives of the different professionals could all be acknowledged. Additionally, we decided to directly address the commonly held myths about depression.

The objective of this paper is to describe the results of a workshop that aims to improve the knowledge of adolescent depression in school staff.

## Materials and Methods

### Design

This was a single-arm trial with a pre-post design. A knowledge questionnaire was administered to participants before and after the workshop.

### Aims and hypotheses

#### General Aim

To evaluate the effectiveness of the workshop “Adolescent depression: What can schools do?” in increasing school staff participant knowledge.

#### Specific Aims

1. To compare the total score of the knowledge questionnaire before and after the workshop.
2. To compare the percentage of correct responses of each question of the knowledge questionnaire before and after the workshop.
3. To compare the total score of the knowledge questionnaire before and after the workshop by different type of staff participants.

### Setting and population

Six workshops were conducted in four cities in Chile [Iquique, Antofagasta, Santiago (two) and Temuco (two)]. School staff from the four cities in Chile was invited to participate in the workshops by e-mail.

### Workshop

The one-session workshops followed a standardized structure, with participatory methodology, led by a child and adolescent psychiatrist (Vania Martínez) and a clinical adolescent psychologist (Daniel Espinosa). Each workshop lasted 4 h and included a PowerPoint presentation, didactic teaching, interactive group exercises and discussion, and film clips. The main topics presented were: relevance, epidemiology, clinical characteristics, etiology, consequences, treatment, prognosis, prevention, school approach, and myths of adolescent depression.

The participants received copies of the PowerPoint presentation and flyers with didactic information, and were introduced to the website www.depresionenadolescentes.cl, where they could review and download more material on adolescent depression.

The authors invite interested readers to visit that website (in the sections “Biblioteca” and “Galería”) to obtain additional Spanish-language resources, or to contact the first author (Vania Martínez) with further information requests.

### Assessment

The Knowledge Questionnaire of Adolescent Depression for School Staff was administered to the participants before and after the workshop. The instrument consists of 26 items, with the alternatives “I agree,” “I disagree,” and “I don’t know.” The “I don’t know” alternative was incorporated in order to avoid random correct responses. The questionnaire includes items about the topics presented in the workshop. Two psychiatrists (Graciela Rojas and Vania Martínez) and an expert in teaching methodology (Rigoberto Marín) developed the questionnaire. Scores can range from 0 to 26. Table 1 shows the questionnaire and the correct responses.

**Table 1:** **Knowledge Questionnaire of Adolescent Depression for School Staff**.

	Item	Correct response
1	In Chile, the annual prevalence of depression in adolescents is not greater than 1%	No
2	At least one in five people, 20 years old of age, have had depression at some point in their lives	Yes
3	The frequency of depression is higher in women than in men after puberty	Yes
4	Recurrent depression that affects adults often begins during adolescence	Yes
5	Approximately 90% of adolescents with depression seek care at a health center	No
6	Depression in adolescence is better defined as a weakness of character than as a disease	No
7	Symptoms must be present for at least 2 weeks to diagnosis depression in adolescents	Yes
8	Depression can be diagnosed in adolescents with a self-report questionnaire answered by the teenager about his or her symptoms	No
9	Blood tests are included as a part of the comprehensive assessment doctors carry out to check for depression in adolescents	Yes
10	Depression should be suspected in adolescents with poor academic performance and repeated absences from school	Yes
11	Depression must be suspected in adolescents with drug abuse	Yes
12	The best initial response after suspecting a teenager of having depression is to talk to him or her	Yes
13	The whole school community must be made aware of a student’s diagnosis of depression	No
14	For the recovery of a teenager with depression, it is preferable that he or she not attending school until symptoms disappear	No
15	The main cause of depression in teenagers is familial dysfunction	No
16	A teenager living through a stressful situation always develops depression	No
17	One of the possible consequences of adolescent depression is suicide	Yes
18	If adolescents manifest a desire to die during school, this should be communicated to their parents or guardians	Yes
19	In all cases of depression, psychosocial interventions and/or psychotherapy should be used for treatment	Yes
20	A teenager with depression and lack of energy should be exempted from physical education class	No
21	If an adolescent has severe depression, his or her parents must choose whether they prefer to start treatment with antidepressant medication or with psychological therapy	No
22	Antidepressants for the treatment of depression in adolescents must be avoided because they produce dependence	No
23	If antidepressant medications are prescribed for an adolescent, they must not be used for longer than 3 months	No
24	The beneficial effects of antidepressant medication begin to be felt after 3–4 weeks	Yes
25	Adolescents who have had depression are not able to 100% recover from this disease	No
26	Depression cannot be prevented because it is an inherited disease	No

### Statistical analyses

Descriptive analysis of the sample was performed. The mean and SD of the number of correct responses before and after the workshop were calculated, and the difference was tested for significance using a *t*-test for paired samples, with unequal variances. When it was not possible to verify or assume normality, the Wilcoxon signed-rank test was performed. The percentage of correct responses and “I don’t know” responses before and after the workshop was calculated for each item to determine the absolute and relative differences. The statistical significance of differences in the percentages of correct responses and “I don’t know” responses was calculated using chi-square, or Fisher’s exact test, when the expected values were 5 or less in any cell. The statistical significance differences in the initial and final total scores between professions were estimated using *t*-test and Wilcoxon sum-rank test depending on the normality assumptions. The results of this analysis are expressed as means, including their respective 95% confidence intervals. All analyses were performed using the statistical software R 3.0.1.

### Ethics

Full ethical approval was obtained from the local Committee (Faculty of Medicine, Universidad de Chile, project number 024-2013). Informed and written consent was obtained from the participants.

## Results

A total of 185 people consented to participate in the trial. Seven participants were excluded because they were not school staff, along with 26 participants who did not complete both questionnaires, resulting in a final sample of 152 school staff. Table 2 shows the main descriptive characteristics of the participants. There were more women than men. The mean age was 35.9 years, with 68.4% of the sample ranging from 23 to 39 years of age. Almost half of the sample was psychologists (44.7%), and almost half worked in public schools (48.7%).

**Table 2:** **Descriptive characteristics of the participants**.

	Female		Male		Total	
*N* (%)	113	(74.3)	39	(25.7)	152	(100)
Age, mean (SD)	35.5	(10.1)	37.0	(10.8)	35.9	(10.3)
Age, *n* (%)
23–29	43	(38.1)	14	(35.9)	57	(37.5)
30–39	36	(31.9)	11	(28.2)	47	(30.9)
40–49	17	(15.0)	7	(17.9)	24	(15.8)
50–59	17	(15.0)	6	(15.4)	23	(15.1)
60	0	(0.0)	1	(2.6)	1	(0.7)
Main role, *n* (%)
Psychologist	51	(45.1)	17	(43.6)	68	(44.7)
Teacher	26	(23.0)	12	(30.8)	38	(25.0)
School counselor	23	(20.4)	4	(10.2)	27	(17.8)
Social worker	8	(7.1)	0	(0.0)	8	(5.3)
Other	5	(4.4)	6	(15.4)	11	(7.2)
Type of school
Public	53	(46.9)	21	(53.8)	74	(48.7)
Private subsidized	54	(47.8)	15	(38.5)	69	(45.4)
Private paid	6	(5.3)	3	(7.7)	9	(5.9)

On average, there were 69.6% (SD 21.3) correct responses on the initial test, and 91.9% (SD 8.0) on the final test. Table 3 summarizes the mean total score of the Knowledge Questionnaire of Adolescent Depression for School Staff and the number and percentage of the participants scoring in four ranges before and after the workshop (pre-test and post-test). The percentage of participants scoring 90% or higher was only 3.9% pre-test, compared to 63.2% post-test. The mean test total score showed statistically significant improvement (*p* = 0.000) from pre-test (18.1) to post-test (23.9).

**Table 3:** **Pre- and post-test total score on the Knowledge Questionnaire Of Adolescent Depression For School Staff**.

Total score range	% Correct	Pre-test		Post-test		
		*n*	%	*n*	%	
0–13	<51	15	9.9	0	0.0	
14–18	51–70	62	40.8	2	1.3	
19–23	71–90	69	45.4	54	35.5	
24–26	>90	6	3.9	96	63.2	

		**M**	**SD**	**M**	**SD**	***p***

Total score		18.1	3.6	23.9	1.8	0.000**

*p*-value: *t*-test for paired sample with unequal variances; ** <0.001.

**Figure 1:**
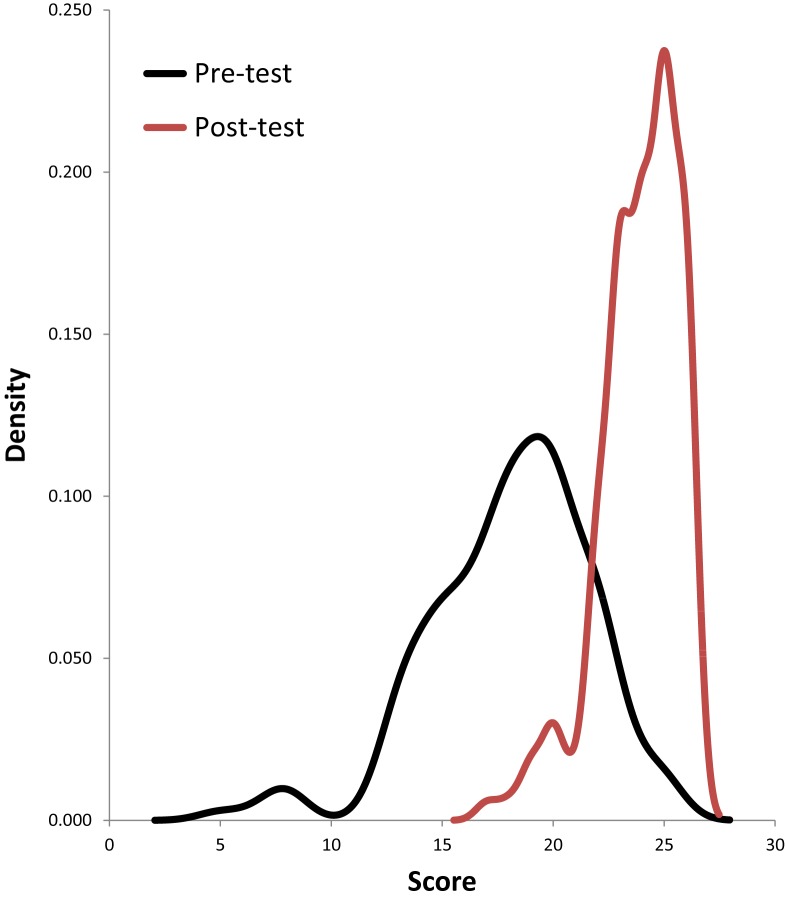
**Distribution of the total score before and after the workshop**.

Figure 1 shows the distribution of the total score before and after the workshop.

Table 4 summarizes the correct responses for each item of the questionnaire. There were statistically significant improvements in 23 of the 26 items from pre-test to post-test.

**Table 4:** **Differences between pre- and post-test correct responses on the Knowledge Questionnaire of Adolescent Depression for School Staff**.

Item	Pre-test correct		Post-test correct		Absolute delta	Relative delta	*p*
	*n*	%	*n*	%	
1	130	86	148	97	12	1.1	0.000**
2	103	68	126	83	15	1.2	0.003*
3	111	73	148	97	24	1.3	0.000**
4	99	65	146	96	31	1.5	0.000*
5	135	89	148	97	9	1.1	0.007*
6	114	75	144	95	20	1.3	0.000**
7	53	35	148	97	63	2.8	0.000**
8	65	43	113	74	32	1.7	0.000**
9	58	38	142	93	55	2.4	0.000**
10	130	86	151	99	14	1.2	0.000**
11	130	86	150	99	13	1.2	0.000**
12	136	89	151	99	10	1.1	0.000**
13	99	65	130	86	20	1.3	0.000**
14	127	84	142	93	10	1.1	0.012*
15	51	34	108	71	38	2.1	0.000**
16	135	89	147	97	8	1.1	0.015*
17	142	93	146	96	3	1.0	0.441
18	147	97	149	98	1	1.0	0.723
19	140	92	147	97	5	1.0	0.134
20	124	82	142	93	12	1.2	0.003*
21	103	68	127	84	16	1.2	0.002*
22	90	59	146	96	37	1.6	0.000**
23	40	26	118	78	51	3.0	0.000**
24	62	41	146	96	55	2.4	0.000**
25	103	68	127	84	16	1.2	0.002*
26.	121	80	142	93	14	1.2	0.001*

*n* = 152; *p*-value: chi-square or Fisher’s exact test (used when the expected values were 5 or less in any cell); * <0.05; ** <0.001.

There were notable increases of correct responses on statements dealing with myths: Item 22 “Antidepressants for the treatment of depression in adolescents must be avoided because they produce dependence” (59–96%) and Item 6 “Depression in adolescence is better defined as a weakness of character, than as a disease” (75–95%).

Items with the greatest change, in terms of relative delta, were: Item 23 “If antidepressant medications are prescribed for an adolescent, they must be used for no longer than three months” (3.0) and Item 7 “Symptoms must be present for at least 2 weeks to diagnosis depression in adolescents” (2.8).

Table 5 also indicates the number of “I don’t know” responses for each item. There were statistically significant decreases in the percentage of “I don’t know” responses for 22 of the 26 items, from pre-test to post-test. Items with the greatest decrease in “I don’t know” responses (absolute delta >30%) referred to depression diagnostic criteria (Item 7), assessment evaluations (Item 9), and anti-depressant medication (Items 23 and 24).

**Table 5:** **Differences between pre- and post-test “I don’t know” responses on the Knowledge Questionnaire of Adolescent Depression for School Staff**.

Item	Pre-test “I don’t know”		Post-test “I don’t know”		Absolute delta	Relative delta	*p*
	*n*	%	*n*	%	
1	19	13	1	1	−12	0.05	0.000**
2	34	22	0	0	−22	0.00	0.000**
3	25	16	0	0	−16	0.00	0.000**
4	33	22	0	0	−22	0.00	0.000**
5	13	9	0	0	−9	0.00	0.001*
6	14	9	1	1	−9	0.07	0.003*
7	51	34	0	0	−34	0.00	0.000**
8	42	28	3	2	−26	0.07	0.000**
9	71	47	2	1	−45	0.03	0.000**
10	2	1	0	0	−1	0.00	0.498
11	8	5	0	0	−5	0.00	0.007*
12	11	7	0	0	−7	0.00	0.001*
13	10	7	1	1	−6	0.10	0.011*
14	16	11	1	1	−10	0.06	0.001*
15	27	18	3	2	−16	0.11	0.000**
16	8	5	0	0	−5	0.00	0.007*
17	2	1	0	0	−1	0.00	0.498
18	2	1	0	0	−1	0.00	0.498
19	5	3	0	0	−3	0.00	0.061
20	19	13	1	1	−12	0.05	0.000**
21	16	11	1	1	−10	0.06	0.001*
22	39	26	1	1	−25	0.03	0.000**
23	88	58	3	2	−56	0.03	0.000**
24	75	49	0	0	−49	0.00	0.000**
25	35	23	0	0	−23	0.00	0.000**
26	26	17	1	1	−16	0.04	0.000**

*n* = 152; *p*-value: chi-square or Fisher’s exact test (used when the expected values were 5 or less in any cell); * <0.05; ** <0.001.

Table 6 shows that all the participants, regardless of their role at school, significantly improved (*p* < 0.05 and *p* < 0.001) the total mean score of the knowledge questionnaire from pre-test to post-test.

**Table 6:** **Pre- and post-test total score of the Knowledge Questionnaire of Adolescent Depression for School Staff by participant role at school**.

Role at school	Pre-test		Post-test		*p*
	*M*	*SD*	*M*	*SD*	
Psychologist	19.8	2.5	24.5	1.3	0.000**
Teacher	16.0	4.5	23.7	2.0	0.000**
School counselor	17.0	3.2	23.3	1.9	0.000**
Social worker	18.3	2.4	23.6	1.1	0.001*
Other	16.9	2.5	22.3	2.5	0.000**

*p*-value: *t*-test for paired sample with unequal variances/Wilcoxon signed-rank test; * <0.05; ** <0.001.

**Figure 2:**
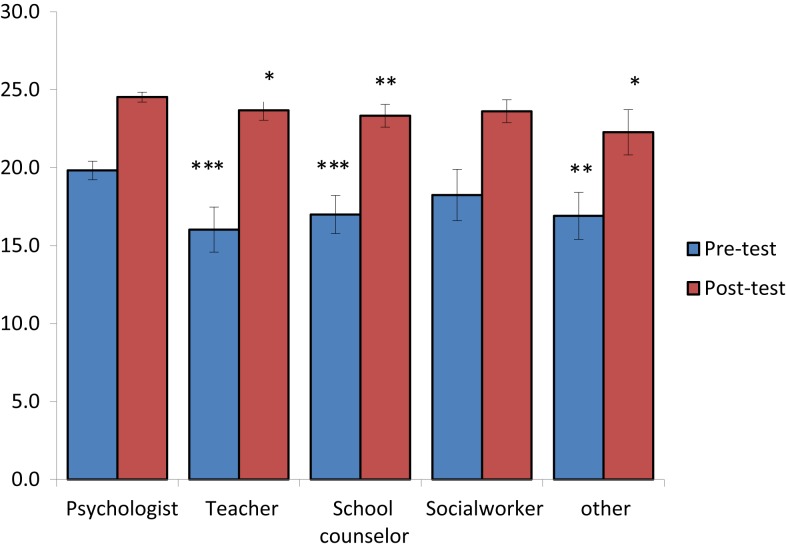
**Before and after performance by professions (psychologist, teacher, school counselor, social worker, and other)**. ***p-value <0.001; **p-value <0.01; *p-value <0.05. *t*-tests for unequal variance/when data were <30 observation in any group was used Wilcoxon Rank-Sum test.

Figure 2 shows that school psychologists performed better on the knowledge questionnaire than the other participants, before and after the workshop. This difference was significant in the pre- and post-test between psychologists and all other participants, except for social workers.

## Discussion

The results of the study show the effectiveness of a brief workshop for school staff in improving their knowledge of adolescent depression. The base-line questionnaire scores can be interpreted as an evaluation of the incorrect ideas commonly held by school staff, which are related to diagnosis (Items 7, 8, and 9), medication (Items 23 and 24), and the etiology of depression (Item 15). After the workshop, the scores of these items improved significantly, and common myths about the disease were dispelled. In fact, only 3 of the 26 items did not show a statistically significant increase (Items 17, 18, and 19), probably because the initial scores were the highest, although they did increase. Moreover, the questionnaire included a number of items pertaining to management of depression in the school setting (Items 13, 14, 18, 20), and participants clarified their doubts with respect to their role in this process.

Although our study found that the school staff had greater knowledge of depression after the workshop, across all discussed areas, we cannot predict how this knowledge will affect the management of depression in the school settings, in terms of early detection of suspected depression cases and referrals to health professionals. Nevertheless, past literature has shown that increased knowledge related to mental health conditions can positively impact the early identification, treatment, and management of the disease (22). This knowledge-focused workshop is the first step, and future studies in Chile should follow-up in schools to evaluate the impact of increased knowledge on the staffs’ detection and referral of depression cases and adolescent student outcomes. Another possibility is that in the future, after the application of this workshop led by a child and adolescent psychiatrist and clinical adolescent psychologist, school psychologists, who scored the highest on the knowledge questionnaire, could take the lead on supporting teachers, administrators, and other school staff in the identification of potential cases.

We found that the tendency of teachers to question the medical model of depression (20) was not seen in this study, perhaps due to the fact that the group of participants was made up of various types of school professionals. This multi-disciplinary group composition in the workshop addressed and considered various perspectives on depression – biomedical, social, and psychological. Future prevention efforts should maintain this comprehensive approach.

One limitation of the study design is that the sample was comprised of school staff who received an open invitation and decided to take part in the workshop – they were not randomly assigned to participate, and thus the participants cannot be considered a representative sample of school staff in Chile. Future studies should use a randomized sampling method and also incorporate a control group and a follow-up period, to assess the long-term effects of the intervention.

This is the first study of its kind in Chile to evaluate a workshop to increase school staff knowledge of adolescent depression. The results are promising, though more studies are required to determine its effects on staff attitudes, timely detection of suspected cases, and improved collaboration with health teams for effective early treatment.

## Author Contributions

VM, DE, and GR conceived the study and were involved in managing and advising the project. RM assisted with teaching methodology. PZ made the statistical analysis of the data. All authors contributed interpreting the data, drafting of the manuscript, and approved the final manuscript.

## Conflict of Interest Statement

The authors declare that the research was conducted in the absence of any commercial or financial relationships that could be construed as a potential conflict of interest.
